# The *Populus* Class III HD ZIP Transcription Factor *POPCORONA* Affects Cell Differentiation during Secondary Growth of Woody Stems

**DOI:** 10.1371/journal.pone.0017458

**Published:** 2011-02-28

**Authors:** Juan Du, Eriko Miura, Marcel Robischon, Ciera Martinez, Andrew Groover

**Affiliations:** 1 Institute of Forest Genetics, Pacific Southwest Research Station, U.S. Forest Service, Davis, California, United States of America; 2 Plant Biology Graduate Group, University of California Davis, Davis, California, United States of America; Iwate University, Japan

## Abstract

The developmental mechanisms regulating cell differentiation and patterning during the secondary growth of woody tissues are poorly understood. Class III HD ZIP transcription factors are evolutionarily ancient and play fundamental roles in various aspects of plant development. Here we investigate the role of a Class III HD ZIP transcription factor, POPCORONA, during secondary growth of woody stems. Transgenic *Populus* (poplar) trees expressing either a miRNA-resistant *POPCORONA* or a synthetic miRNA targeting *POPCORONA* were used to infer function of *POPCORONA* during secondary growth. Whole plant, histological, and gene expression changes were compared for transgenic and wild-type control plants. Synthetic miRNA knock down of *POPCORONA* results in abnormal lignification in cells of the pith, while overexpression of a miRNA-resistant *POPCORONA* results in delayed lignification of xylem and phloem fibers during secondary growth. *POPCORONA* misexpression also results in coordinated changes in expression of genes within a previously described transcriptional network regulating cell differentiation and cell wall biosynthesis, and hormone-related genes associated with fiber differentiation. *POPCORONA* illustrates another function of Class III HD ZIPs: regulating cell differentiation during secondary growth.

## Introduction

Secondary vascular development involves the coordination of several developmental processes, including the patterning of secondary vascular tissues and differentiation of complex cell types [1]. In the model forest tree genus *Populus*, the cambium is typically believed to contain a single layer of initials, from which xylem mother cells are derived towards the inside (towards the pith) of the stem and phloem mother cells towards the outside (towards the epidermis) [2]. Xylem and phloem mother cells divide one or more times before differentiating into cell types within the secondary xylem (wood) or secondary phloem (inner bark), respectively. Conspicuous, lignified phloem fibers differentiate in the periphery of the phloem. In addition to these vertically oriented tissue systems, the cambium contains ray initials, which ultimately produce rays that transverse secondary xylem and phloem and transport water and solutes radially in the stem. The resulting woody stem is thus the result of coordination between radial patterning processes that produce tissues in appropriate positions, and differentiation of secondary vascular cell types within those tissues.

Although still poorly defined, insights into mechanisms regulating cell differentiation during secondary growth are emerging, and have been assisted by the observation that at least some of the key regulatory genes that regulate shoot apical meristems and primary vascular development are also expressed during secondary growth [3], [4], [5]. For example, Class I KNOX genes are well characterized for their roles in regulating stems cells and cell differentiation in shoot apical meristems, but they also play important roles in negatively regulating the differentiation of cambial and cambial daughter cells [6], [7]. In contrast, NAC-domain containing transcription factors have been identified that promote differentiation of vessel elements including VASCULAR-RELATED NAC-DOMAIN (VND), NAC SECONDARY CELL WALL THICKENING (NST), and SECONDARY WALL-ASSOCIATED NAC DOMAIN PROTEIN (SND) proteins, likely through direct regulation of MYB-class transcription factors that in turn regulate expression of cell differentiation and cell wall-related genes {reviewed in [8]}. For example, overexpression of *VND6* or *VND7* results in ectopic differentiation of vessel elements in *Arabidopsis thaliana*, which is accompanied by overexpression of MYB genes (including *MYB46*, *MYB63*, *MYB83*, *MYB85*, and *MYB103*) and downstream genes encoding enzymes involved in cell wall biosynthesis and vessel differentiation (including *CESA4*/*IRX5*, *CESA7*/*IRX3*, *CESA8*/*IRX1*, *IRX8*, *IRX10*, *CCoAOMT7*, *IRX12*/*LAC4*, and *XCP1*) [9]. However, this putative transcriptional network has not been evaluated during secondary growth.

While putative genes and mechanisms regulating cell differentiation are becoming better described, less is known about regulation of tissue patterning during secondary growth. In *Arabidopsis thaliana*, the Class III HD ZIPs comprise a small family of five genes, *PHABULOSA* (*PHB*), *PHAVOLUTA* (*PHV*), *REVOLUTA* (*REV*), *ATHB8*, and *CORONA*/*ATHB15* (*CNA*). Combinations of loss of function mutants, gain of function mutants, and cross-complementation studies indicate overlapping yet distinct roles for *A. thaliana* Class III HD ZIPs [10], [11], [12], [13]. Functional differences among Class III HD ZIPs are likely attributable both to differences in expression patterns, as well as differences in protein function [10]. *PHB*, *PHV*, and *REV* form a subclade, and have been implicated in acting antagonistically with KANADI transcription factors to regulate polarity and patterning of lateral organs and vascular bundles [12], [13], [14], [15], [16], [17]. *CORONA*/*ATHB15/INCURVATA4* (*CNA*) and *ATHB8* form the second subclade of *A. thaliana* Class III HD ZIPs. *ATHB8* is expressed in procambial cells in embryos and developing organs, and *ATHB8* expression is induced by exogenous auxin [18]. Overexpression of *ATHB8* is associated with precocious differentiation of xylem and lignification of cell types that are normally not lignified [19]. All known land plant Class III HD ZIPs contain a binding site for negative regulation posttranscriptionally by miRNA165/166 [20]. Mutations that abolish the miRNA binding sequence without changing amino acid sequence result in dominant phenotypes for Class III HD ZIPs [12], [13], [21], [22], [23], [24].

*CNA* is expressed in provascular cells of leaves and roots [25], in the shoot meristem, in floral meristems, and in ovules [11]. *A Zinnia elegans* ortholog of *CNA*, *ZeHB-13*, is expressed in leaf provascular cells and developing xylem parenchyma of leaf vascular bundles, but is not expressed in mature leaves [25]. In *A. thaliana*, overexpression of a miRNA-resistant *ATHB15* results in moderate dwarfing, upcurling of leaves, and drastic reduction in xylem and lignified interfascicular tissues [21]. Similar phenotypes are seen in mutants carrying semidominant alleles of CNA with a single point mutation abolishing normal miRNA regulation [26], [27], [28]. Antisense *ATHB15* transgenics are severely dwarfed, and display expansion of xylem and interfascicular tissues, and lignification into the pith of stems [21]. In contrast, EMS-induced *cna* mutants show slightly increased shoot apical meristem size [11] but no other dramatic phenotypes [11], [27], and RNAi suppression of CNA transcripts was reported not to result in obvious phenotypic changes [27]. Interestingly, *cna* is a dramatic enhancer of meristem size in *clavata1/2/3* mutants, indicating that *CNA* may function with *CLV* genes to promote organ formation [11]. However, neither the genes directly regulated by CNA nor the biological functions influenced by CNA have been identified, and CNA function during secondary growth in plants has not been examined.

Class III HD ZIP transcription factors are evolutionarily ancient and found in all major lineages of land plants [17], [20], [29]. Importantly, Class III HD ZIPs predate evolution of vascular tissues and adaxial polarity of lateral organs [29], suggesting that many of the functions assigned to Class III HD ZIPs in angiosperms are derived. Ancestral functions of Class III HD ZIPs could include regulation of apical or meristematic growth [20], [29], and auxin transport [30]. While Class III HD ZIPs roles during secondary growth have not been characterized, they are known to be expressed during secondary growth from microarray analysis of wood forming tissues in *Populus* [5], [31], [32]. Class III HD ZIPs are thus candidates for regulating fundamental developmental processes acquired during the evolution of secondary growth.

We report here the cloning and characterization of a *Populus CNA* ortholog, *POPCORONA* (*PCN*). We defined roles for PCN during secondary growth by examining *PCN* expression and mutant phenotypes in transgenic *Populus* expressing either an artificial miRNA targeting *PCN* transcripts or overexpressing a miRNA-resistant form of *PCN*. Together, our results suggest that *PCN* regulates development of secondary vascular tissues, potentially through regulation of transcriptional modules associated with cell differentiation and/or hormone-mediated processes.

## Results

### *POPCORONA* encodes a Class III HD ZIP transcription factor

**Figure 1:**
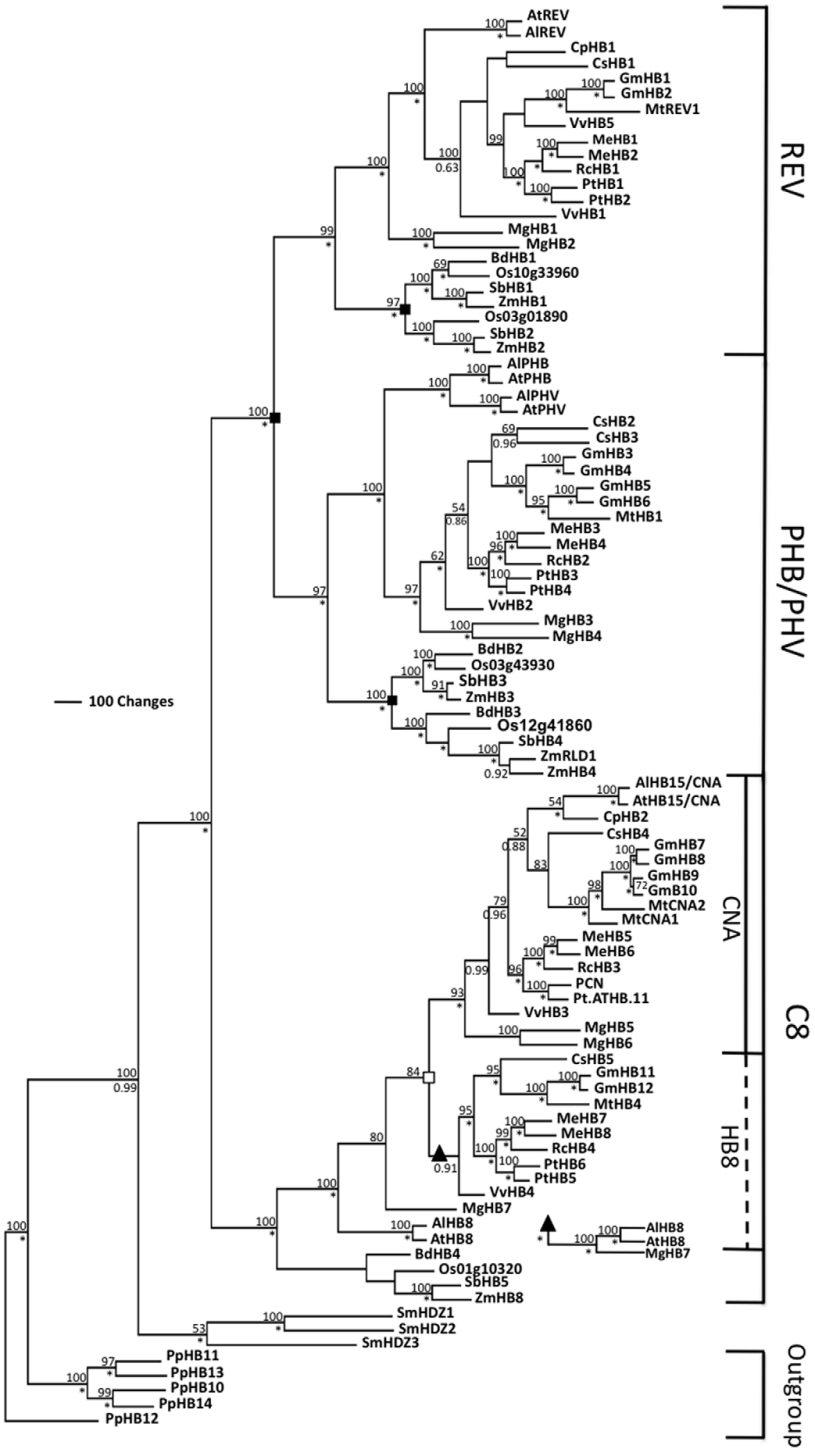
Phylogenetic relationships among Class III HD ZIP gene family in land plants determined using maximum parsimony analysis. Bootstrap support values above 50% are presented above branches and Bayesian support values above 0.50 are presented below branches, where * indicates maximum 1.00 support. Black squares indicate major duplication events, while the empty square represents evidence of a duplication event without bootstrap support. Major clades are presented by longitudinal lines to the right of the tree, where solid lines represent fully supported monophyletic clades (PHB, C8, and CNA) and dashed lines indicate clade supported by Bayesian, but not bootstrap support. Black triangles represent where the *AtHB8, AlHB8, MgHB7* clade is supported according to Bayesian analysis. Species abbreviations: *At*, *Arabidopsis thaliana; Bd, Brachypodium distachyon; Al, Arabidopsis lyrata; Cp, Carica papaya; Cs, Cucumis sativus; Gm, Glycine max; Me, Manihot esculenta; Mt, Medicago truncatula; Mg, Mimulus guttatus; Os, Oryzas sativa; Pt, Populus trichocarpa; Pp, Physcomitrella patens; Rc, Ricinus communis; Sm, Selanginella moellendorffii, Sb, Sorghum bicolor; Vv, Vitis vinifera; Zm, Zea mays.*

The *POPCORONA* (*PCN*) gene (also called Pt-ATHB.12; Joint Genome Institute Populus v.1.1 gene model fgenesh4_pm.C_LG_I000560; Phytozome Populus v2.0 gene model POPTR_0001s18930: GenBank XM_002299699) was amplified from cDNA of the sequenced *Populus trichocarpa* individual [33] based on similarity to the *Arabidopsis thaliana ATHB15*/*CORONA* (Materials and Methods). To determine the relationship of *PCN* to other Class III HD ZIPs, a phylogenetic analysis of Class III HD ZIP sequences from whole-genome sequencing projects was undertaken (Materials and Methods). Maximum parsimony analysis from the nucleotide sequence alignment of the coding sequences of Class III HD ZIPs found in 17 plant species and deduced from amino acid sequence alignments yielded a single tree, with clade support from Bayesian and bootstrap analysis (Fig. 1). Sequences from *Physcomitrella patens,* a moss, were used as outgroups to root the tree. Sequences from the lycophyte representative, *Selanginella moellendorffii,* formed a clade (53% bootstrap, 1.00 Bayesian) sister to sequences from the angiosperm taxa, which formed a strongly supported (100% bootstrap, 1.00 Bayesian) clade. These results all reflect previously reported Class III HD ZIP gene family relationships [17], [20]. Among the angiosperm sequences, three strongly supported clades were formed, REV, PHB/PHV, and C8, which is in agreement with previous reports [17]. REV and PHB/PHV clades are sister to each other forming a larger clade that is sister to C8. Relationships within each of the three clades are generally consistent with currently accepted ideas about angiosperm phylogeny [34]. Our analysis includes four representative species from the grass lineage; *O. sativus, Z. mays, B. distachyon,* and *S. bicolor*, allowing further details in the divergence of homologues within this monocot lineage. Sequences from these four species form monophyletic clades separate from the eudicot species. Within these clades two duplication events appear to have occurred after the monocot-eudicot split. This duplication likely represents the grass whole genome duplication event [35], [36].

The major duplication events previously reported are mostly in agreement with our phylogenetic tree, with a few exceptions. Our phylogeny does not include representatives of species within the ferns, basal angiosperms, or gymnosperms, and therefore our analysis cannot dispute or support duplication events that were suggested previously to have occurred in these lineages. In our parsimony tree (Fig. 1), bootstrap supports *AtHB8* and *AlHB8* (100% bootstrap), and *MgHB7* (80% bootstrap) as sister to the rest of the CNA/HB8 eudicot clade, while Bayesian analysis supports them within the HB8 clade (1.00 Bayesian). Supporting *AtHB8, AlHB8,* and *MgHB7* within the HB8 clade is evidence for a duplication event resulting in the HB8 and CNA clades, as previously reported (Prigge and Clark, 2006). Therefore, our parsimony and Bayesian analysis are not in agreement with their placement, and only partially support previous suggestions of a duplication event in C8 clade. This inconsistency may be an artifact of limited and uneven sampling, as discussed above.

Within *Populus*, each Class III HD ZIP is represented by two paralogs (Fig. 1), reflecting the genome duplication event whose signature was found in analysis of the *P. trichocarpa* genome [33]. Independent duplication events are present in the PHV/PHB clade in the *A. thaliana* and *Populus* lineages (Fig. 1). Two paralogs of *A. thaliana ATHB15*/*CNA* are present, including *PCN* and Pt-ATHB.11 (Phytozome *Populus* v2.0 gene model POPTR_0003s04860; Joint Genome Institute *Populus* v1.1 gene model estExt_fgenesh4_pg.C_LG_III0436).

### *PCN* is expressed during secondary growth

**Figure 2:**
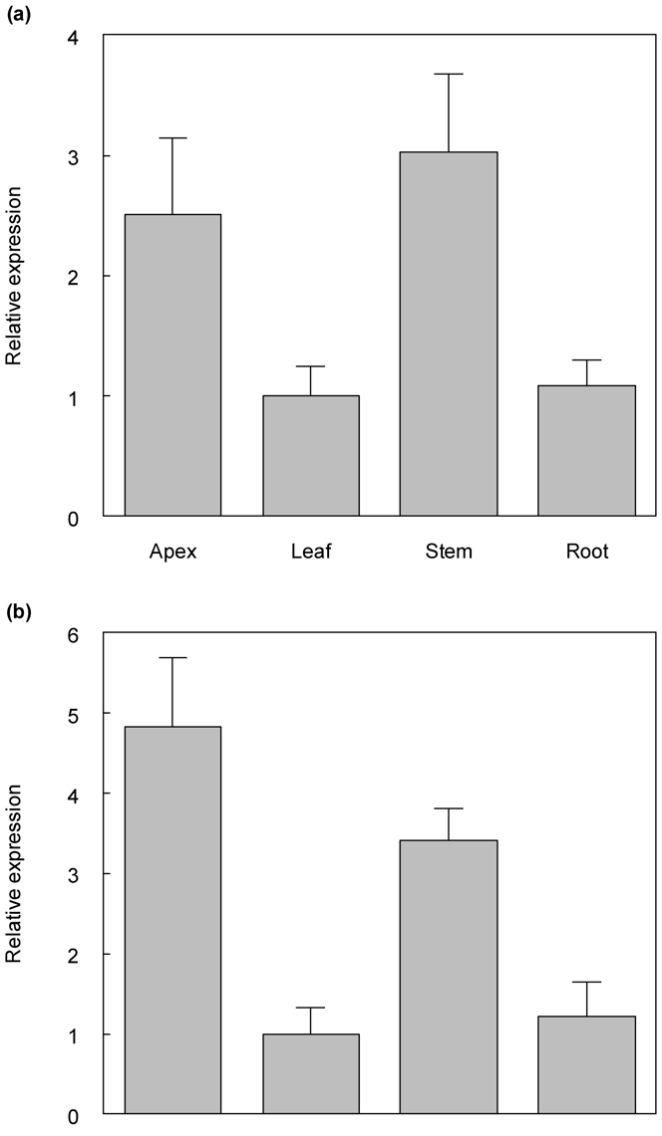
Expression of *PCN* (Fig. 2a) and *PCN* paralog Pt-ATHB.11 (Fig. 2b) in organs, as assayed by Quantitative Real Time PCR. Relative expression of *PCN* and paralog Pt-ATHB.11 in apices, leaves, roots, and stem was determined using Quantitative Real Time PCR (QRT-PCR) of two month old tissue culture grown *Populus tremula x alba*. *PCN* and paralog Pt-ATHB.11 are expressed in all tissues assayed, and are highly expressed in shoot apexes and stem tissue with active cambium. Stem tissue samples were confirmed to have a vascular cambium by phloroglucinol staining of secondary xylem. Relative expression (Mean ± SE) was calculated from triplicate QRT-PCR reactions of independent RNA samples prepared from different trees.

*PCN* is expressed in shoot apices, leaves, stems, and roots of young *Populus* plants. Transcript levels were determined for organs of tissue culture-grown *Populus alba* x *tremula* using quantitative real time PCR (Materials and Methods). Primers amplifying *PCN* transcripts revealed highest expression in stems and apices, with lower but significant expression in leaves and roots (Fig. 2a). Primers amplifying a *PCN* paralog, Pt-ATHB.11, showed similar expression patterns for this gene, although expression was slightly higher in apices relative to stem tissues (Fig. 2b).

**Figure 3:**
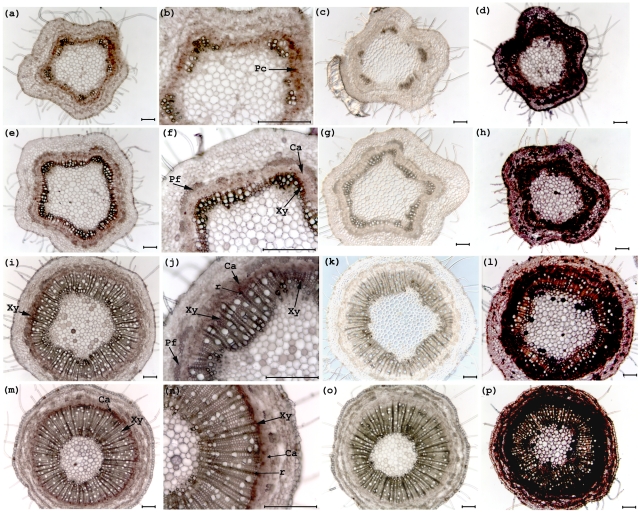
Expression of *PCN* during *Populus* stem development revealed by whole mount *in situ* hybridization. Antisense *PCN* (first and second columns), sense negative control (third column), and positive control (fourth column) probes were hybridized to stem sections from two month old tissue culture grown trees. (a) Section from first elongating internode hybridized with antisense *PCN* probe. *PCN* is expressed broadly during primary growth, with strongest expression associated with procambium. (b) Higher magnification of first elongating internode hybridized with antisense *PCN* probe. (c) Section from first elongating internode hybridized with sense *PCN* probe (negative control), showing minimal background hybridization. (d) Section from first elongating internode hybridized with antisense pop50S probe (positive control). (e) Section from the fourth internode, hybridized with antisense *PCN* probe. *PCN* is expressed broadly in the cambial zone, and strongly in differentiating xylem. (f) Higher magnification of (e). (g) Section from the fourth internode hybridized with negative control sense *PCN* probe. (h) Section from fourth internode hybridized with positive control antisense pop50S probe. (i) Section from seventh internode hybridized with antisense *PCN* probe. *PCN* expression is mostly associated with differentiating xylem cells and lightly in cambial zone. (j) Higher magnification of (i). (k) Section from seventh internode hybridized with sense *PCN* probe (negative control). (l) Section from seventh internode hybridized with positive control antisense pop50S probe. (m) Section from the base internode hybridized with antisense *PCN* probe. *PCN* expression is largely limited to the differentiating xylem cells and cambial zone. (n) Higher magnification of (m). (o) Section from the base internode hybridized with sense *PCN* probe (negative control). (p) Section from the base internode hybridized with positive control antisense pop50S probe. Cambial zone (Ca), Phloem fiber (Pf), Procambium (Pc), Ray (r), Xylem (Xy), Bar = 100 µm.

*PCN* is expressed broadly in the cambial zone and xylem of *Populus* shoots. Whole mount *in situ* hybridization was used to visualize *PCN* expression in tangential sections from different developmental stages of *Populus* stems (Materials and Methods). During primary growth and the transition to secondary growth, *PCN* is expressed broadly in the cambial zone and in developing xylem (Fig. 3a,b). Later in development, phloem fiber differentiation becomes evident (Fig. 3e,f) and weak signal is seen outside of the cambial zone at this stage in the developing phloem, including the phloem fibers (Fig. 3e,f). Moving further down the stem into developmentally older tissues, *PCN* expression is maintained within developing xylem, and is most pronounced in rays (Fig. 3 i,j). At the base of the stem, strongest expression is found in the cambial zone, with reduced expression in the secondary xylem (Fig. 3m,n). However, expression is still pronounced within the rays traversing the secondary xylem (Fig. 3n). In comparison to sense-probe negative controls (Fig. 3c,g,k,o), the in situ staining of experimental sections with the *PCN* antisense probe is specific and has relatively low background. However, based on comparison of *PCN* antisense and sense negative control sections we cannot exclude the possibility of low *PCN* expression, cross hybridization to related genes, or diffusion of the stain in epidermis, pith cells, or other tissues/cell types. Differential staining caused by differences in cytoplasmic density of cell types is revealed by an antisense probe for a presumably ubiquitously expressed gene encoding a 50S ribosomal protein (Fig. 3d,h,l,p).

### Misregulation of *PCN* results in whole plant phenotypes

**Figure 4:**
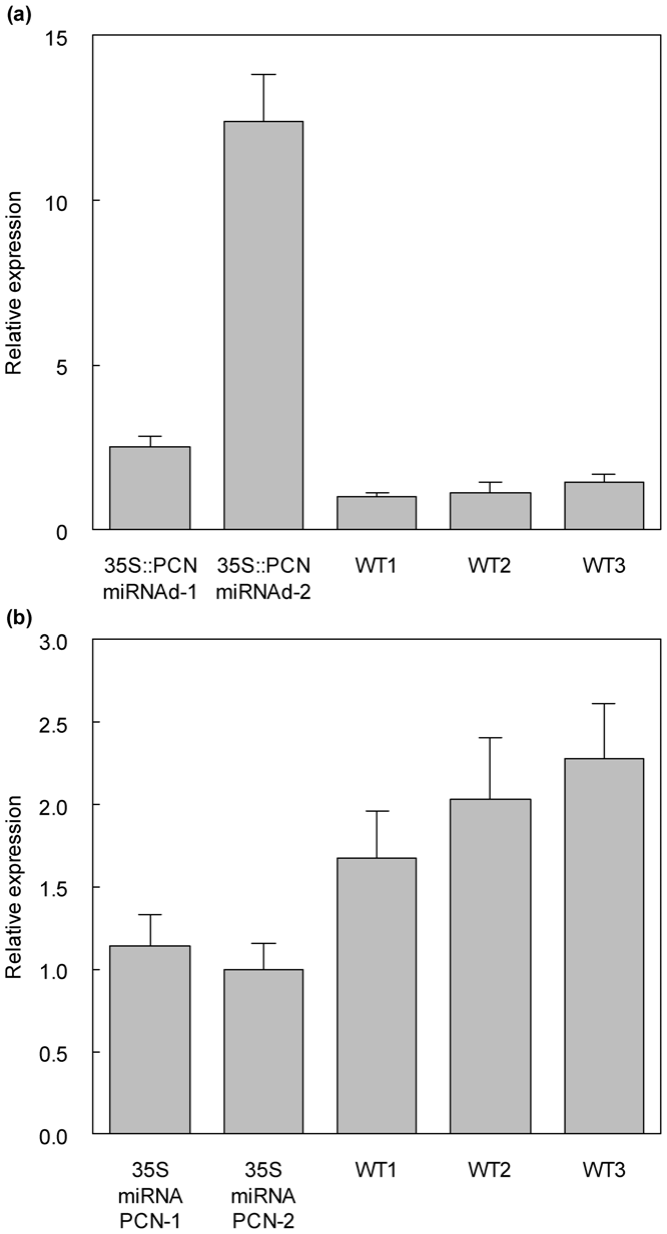
*PCN* expression levels in *PCN 35S::PCN-miRNAd* gain of function and *35S::miRNA-PCN* knockdown transgenic plants relative to wild-type controls. *PCN* expression levels were detected by Quantitative Real Time PCR (Materials and Methods). Relative expression levels (mean ± SE) were calculated from triplicate qRT-PCR reactions of independent RNA samples for each transgenic and the wild-type prepared from different batches of two month-old plants. T test (P<0.05) comparison showed significant differences of expression in all transgenics compared to the wild-types. (a) Comparison *PCN* transcripts in wild-type and *35S::PCN-miRNAd* gain of function plants. (b) Comparison *PCN* transcripts in wild-type and *35S::miRNA-PCN* plants.

To examine the function of *PCN* during plant development, the *Populus* clone INRA 717-IB4 (*Populus alba* x *P. tremula*) was transformed with recombinant DNA constructs to either up or down-regulate *PCN* transcript levels. To upregulate *PCN* transcript levels, a *PCN* cDNA was modified to change the miRNA165/166 recognition sequence without changing the amino acid sequence when translated into protein, thus making the transcript transparent to negative regulation by miRNA165/166 (Materials and Methods). This cDNA was cloned behind a 35S cauliflower mosaic virus promoter in a T-DNA vector to create construct 35S::PCN-miRNAd, and transformed into *Populus* using an *Agrobacterium*-based method (Materials and Methods). To down-regulate *PCN* transcripts levels, an artificial miRNA [37], [38] was designed to target both *PCN* and its paralog, Pt-ATHB.11, and cloned behind the 35S promoter in a T-DNA vector to create construct 35S::miRNA-PCN, which was then transformed into *Populus* (Materials and Methods). Two plants independently transformed with 35S::PCN-miRNAd, and two plants independently transformed with 35S::miRNA-PCN were recovered and used in the analyses here. Quantitative Reverse Transcriptase Polymerase Chain Reaction (Q-RT-PCR) analysis of the transformants and wild-type controls showed that for plants transformed with 35S::PCN-miRNAd, one line (35S::PCN-miRNAd-4-1) had approximately two-fold increase of *PCN* transcript abundance relative to controls, while line 35S::PCN-miRNAd-4-3 had over twelve-fold increase in *PCN* transcript abundance (Fig. 4a). Only modest downregulation of *PCN* transcript abundance was found for artificial miRNA lines (up to approximately two-fold reduction in line 35S::miRNA-PCN-3-4), indicating the artificial miRNA was only partially effective in reducing *PCN* transcripts (Fig. 4b).

**Figure 5:**
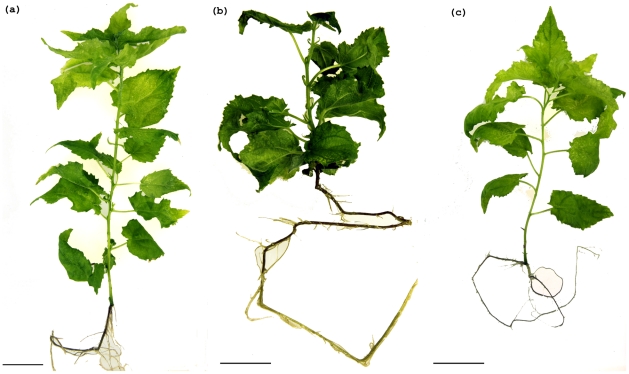
Phenotypes of *PCN 35S::PCN-miRNAd* gain of function and *35S::miRNA-PCN* knockdown plants compared to wild-type controls. (a) Wild-type plants (2 months old). (b) *PCN 35S::PCN-miRNAd* gain of function (2 months old) plants have changes to plant architecture, shorter plants length, darker green color in leaf. (c) *35S::miRNA-PCN* knockdown plants (2 month old) have no strong differences from the wild-type. Bar = 2.5cm.

Compared with matched wild-type controls (Fig. 5a), 35S::PCN-miRNAd-4-3 plants have shorter internodes, darker green leaves, and large stipules (Fig. 5b). The mutants did not present any obvious polarity defects, barren axils, or root phenotypes. Plants transformed with 35S::miRNA-PCN did not show any consistent whole plant phenotypes in culture, perhaps reflecting that the modest decrease in transcript abundance for *PCN* and its paralog in these plants, or that loss of *PCN* function may not result in a strong phenotype.

### Misregulation of *PCN* alters secondary growth

**Figure 6:**
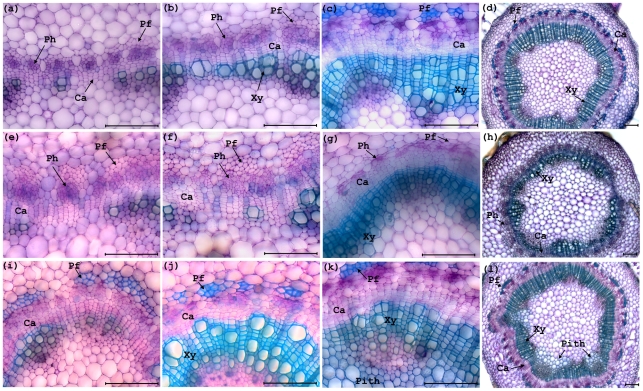
Transverse sections of stems from two month old wild-type and *35S::PCN-miRNAd* gain of function and *35S::miRNA-PCN* knockdown *Populus*. (a) Section from fourth internode of wild-type *Populus* stem during primary growth. (b) Section from seventh internode of wild-type *Populus* stem during transition to secondary growth, showing secondary xylem tissue formation. (c) Section from bottom internode of wild-type *Populus* stem showing secondary phloem fibers and secondary xylem tissue. (d) Lower magnification view of bottom internode of wild-type stem. (e) Section from fourth internode of *35S::PCN-miRNAd* gain of function *Populus* stem during primary growth, showing increased cambium cell layers. (f) Section from seventh internode of *35S::PCN-miRNAd* gain of function *Populus* stem during transition to secondary growth, showing delayed secondary xylem formation. (g) Section from bottom internode of *35S::PCN-miRNAd* gain of function *Populus* stem showing no lignified phloem fibers formation and decreased xylem tissue. (h) Lower magnification of section from bottom internode of *35S::PCN-miRNAd* gain of function *Populus* stem showing no lignified phloem fibers formation and decreased xylem tissue. (i) Section from fourth internode of *35S::miRNA-PCN* knockdown *Populus* stem showing early formed lignified phloem fibers and xylem cells by comparing with the wild-type. (j) Section from seventh internode of *35S::miRNA-PCN* knockdown *Populus* stem showing increased secondary phloem fibers and xylem tissue formation by comparing with the wild-type. (k) Section from bottom internode of *35S::miRNA-PCN* knockdown *Populus* stem showing ectopic lignifications in pith cells. (l) Lower magnification of section from bottom internode of *35S::miRNA-PCN* knockdown *Populus* stem showing ectopic lignifications in pith cells. Cambial zone (Ca), Phloem (Ph), Phloem fiber (Pf), Xylem (Xy), Bar = 100 µm.

Wild-type *Populus* make a gradual transition from primary to secondary growth. Under our culture conditions, wild-type *Populus* clone INRA 717-IB4 (*Populus alba* x *P. tremula*) is already transitioning to secondary growth by the fourth elongating internode from the apex, as seen in stem cross sections stained with toluidine blue (Fig. 6). By the seventh internode, secondary xylem characterized by cell files derived from the cambial initials is apparent (Fig. 6a). Nascent phloem fibers are apparent by the seventh node but are not yet highly lignified. At the base of the stem, cell files of lignified secondary xylem can be seen emanating from the cambial zone, and fully differentiated and lignified phloem fibers are seen at the periphery of the phloem (Fig. 6c). At lower magnification (Fig. 6d), the radial organization of the stem is seen, with successive layers of pith, secondary xylem, cambial zone, secondary phloem and phloem fibers, cortex, and epidermis.

In 35S::PCN-miRNAd-4-3 plants, cell files within the cambial zone are more readily apparent than in wild-type control plants at the fourth internode (Fig. 6e). The premature appearance of differentiating phloem fibers also distinguishes these stems from the wild-type, although the fibers are not lignified (Fig. 6e). Internode seven of 35S::PCN-miRNAd-4-3 plants is characterized by a distinct cambial zone, modest secondary xylem, and unlignified cells presumed to be differentiating phloem fibers (Fig. 6f). A section from the base of the stem shows relatively well-developed secondary xylem, but is largely lacking the lignified phloem fibers that conspicuously occur in the periphery of the phloem of wild-type plants. Thus, although phloem fiber differentiation appears to initiate prematurely in 35S::PCN-miRNAd plants, they do not complete normal differentiation and lignification.

**Figure 7:**
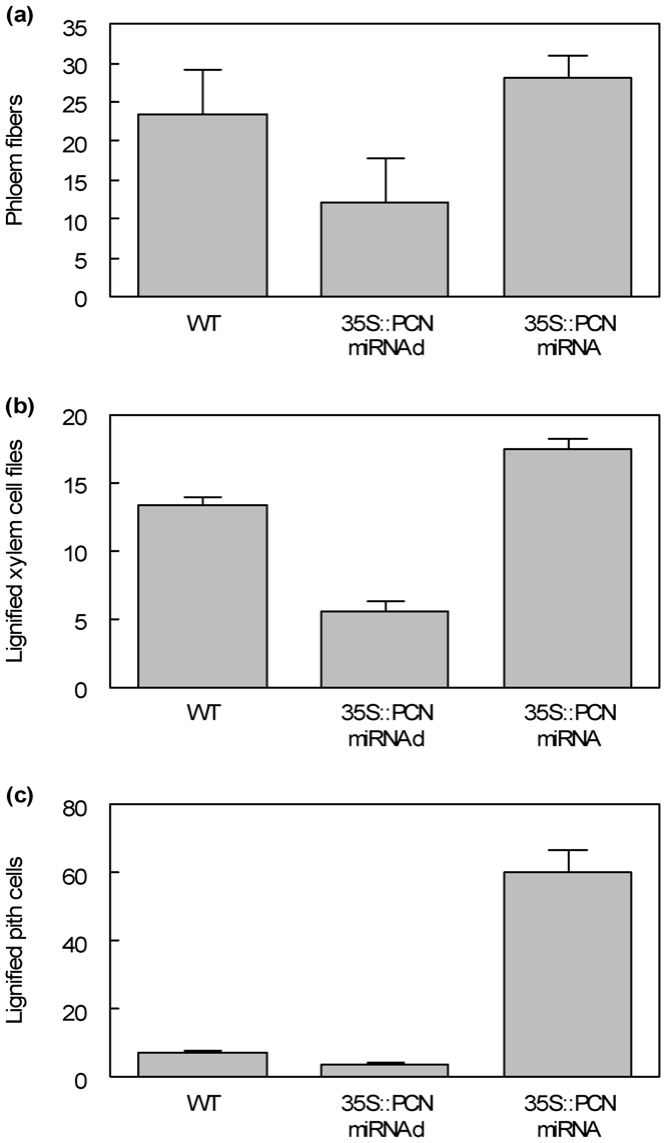
Quantification of phenotypes in bottom internode of *35S::PCN-miRNAd* gain of function and *35S::miRNA-PCN* knockdown transgenics. (a) Comparison of number of phloem fibers in the bottom internodes of wild-type, *35S::PCN-miRNAd* gain of function and *35S::miRNA-PCN*. (b) Comparison of number of lignified xylem cell layers in the bottom internodes of wild-type, *35S::PCN-miRNAd* gain of function and *35S::miRNA-PCN*. (c) Comparison of number of lignified pith cells in the bottom internodes of wild-type, *35S::PCN-miRNAd* gain of function and *35S::miRNA-PCN*. Relative expression levels (mean ± SE) were calculated from three cross-sections of the bottom internodes of three independent wild type plants, three *miRNAd* gain of function transgenics, three 35S::PCN-miRNAd transgenics prepared from different batches of two month-old plants.

As mentioned, 35S::miRNA-PCN plants had only a modest reduction in *PCN* transcript abundance (Fig. 4), and no whole plant phenotype was apparent (Fig. 5). However, cross sections revealed subtle defects in stem development. At the fourth internode from the apex, lignified phloem fibers are already apparent in 35S::miRNA-PCN plants, while they are lacking at this stage of development in the wild-type (Fig. 6I). Also at this position, cambium activity is evident as cell files within the cambial zone, and modest amounts of secondary xylem have formed. By internode seven, 35S::miRNA-PCN plants have more abundant secondary and noticeably more highly lignified phloem fibers than the wild-type (Fig. 6j). Sections through the bottom of the stem are similar to the wild-type, except that there is abnormal lignification of some cells within the pith (Fig. 6 k,l). These abnormally lignified cells are primarily found adjacent to the position of primary vascular bundles (Fig. 6k,l). In summary, misexpression or down-regulation of PCN results in differences in the number of phloem fibers, number of xylem cell layers, and number of lignified pith cells, as quantified in Fig. 7.

### Misregulation of *PCN* changes expression of genes associated with gene regulation, hormones and cell differentiation

To begin to understand the biological processes and genes regulated directly or indirectly by *PCN* during secondary growth, global transcript abundance was assayed using microarrays for stems from wild-type control and 35S::PCN-miRNAd-4-3 plants (Materials and Methods). RNA was isolated from stems of four plants each of 35S::PCN-miRNAd-4-3 and matched wild-type controls (Materials and Methods). The RNA quality and quantity was assayed with Agilent Bioanalyzer before being labeled and hybridized to Affymetrix *Populus* whole transcriptomes gene chips (Materials and Methods). The resulting data were normalized and analyzed using dChip software (http://biosun1.harvard.edu/complab/dchip/) to identify genes with statistically differential expression between the wild-type and 35S::PCN-miRNAd stems (Materials and Methods). Data are available as accession GSE19467 through NCBI GEO (http://www.ncbi.nlm.nih.gov/geo/).

Of the approximately 45,000 genes in the *Populus* genome [33], 237 genes showed statistically significant two-fold or greater differential transcript abundance between the wild-type and 35S::PCN-miRNAd stems (see Supplemental Table S3 for analysis of all probes with statistically different transcript levels). Of these genes, those belonging to three biological categories (transcription, hormone-related, and cell wall-related) are discussed here in more detail with the idea that they are potentially associated with the function of *PCN* and the phenotype of *PCN* transgenics. It should be noted that presumably only a subset of these genes may be direct targets of PCN.

Sixteen genes encoding transcription factors from ten different families are misregulated in 35S::PCN-miRNAd stems (Table 1). Interestingly, this group includes *Populus* orthologs of *A. thaliana* transcription factors that have been implicated in a transcriptional network controlling vascular cell differentiation and lignification. SECONDARY CELL WALL-ASSOCIATED NAC DOMAIN (SND) and VASCULAR RELATED NAC DOMAIN (VND) proteins have been shown to positively regulate expression of specific MYB transcription factors [9], [39], [40], [41]. In 35S::PCN-miRNAd stems, transcript levels of *Populus* orthologs of NAC7/VND4 (gw1.III.864.1) and SND2 (eugene3.01240095) are upregulated, as are orthologs of previously proposed VND/SND targets MYB83 (gw1.IX.3293.1), MYB85 (gw1.IX.3293.1), MYB52 (eugene3.00120054) [9], [39]. Additionally, a *Populus* ortholog (fgenesh4_pm.C_scaffold_66000095) of *XYLEM ENDOPEPDIDASE 1* (*XCP1*) as well as categories (cellulose synthesis-related, lignin-related) and specific orthologs of cell wall-related genes (including *IRREGULAR XYLEM3* and *IRX8* orthologs) that are targets of these MYB transcription factors [39] are misregulated in 35S::PCN-miRNAd stems (Table 2).

**Table 1:** Transcription factors mis-regulated in PCN transgenics.

JGI Gene Accession^a^	Fold Change^b^	Arabidopsis^c^	Definition Line^d^
grail3.0041013401	4.25	AT1G71692	AGAMOUS-like 12 (AGL12)
gw1.IX.3293.1	4.16	AT3G08500	MYB domain protein 83 (MYB83)
gw1.III.861.1	4.01	AT4G22680	MYB domain protein 85 (MYB85)
eugene3.00700057	3.62	AT2G30580	putative C3HC4 zinc finger protein
gw1.III.864.1	3.47	AT1G12260	NAM protein, NAC7/VND4
eugene3.00120054	3.36	AT1G17950	MYB domain protein 52 (MYB52)
*grail3.0001068602	3.24	AT1G10200	transcription factor LIM
estExt_fgenesh4_pg.C_570199	2.99	AT4G36740	A. thaliana homeobox protein 40 (ATHB40)
gw1.V.991.1	2.96	AT3G22830	heat shock transcription factor A6B (HSFA6B)
eugene3.01310019	2.7	AT1G09540	MYB domain protein 61 (MYB61)
eugene3.01240095	2.65	AT4G28500	NAM protein, SND2
gw1.II.2517.1	−2.83	AT3G60530	GATA transcription factor 4 (GATA4)
gw1.XI.848.1	−3.26	AT1G01030	DNA-binding protein
fgenesh4_pg.C_LG_XIV000644	−4.28	AT1G67030	zinc finger protein (ZFP6)
*gw1.I.8677.1	−4.96	AT4G23750	AP2 domain transcription factor, CRF2/TMO3
estExt_Genewise1_v1.C_LG_XIV3374	−7.1	AT4G22070	WRKY DNA-binding protein 31 (WRKY31)

a. Accession number assigned by the Joint Genome Institute (http://genome.jgi-psf.org).

b. Fold Change is expressed as the ratio of gene expression in *PCN* gain of function transgenenics to wild type control.

c. Accession number of the best Arabidopsis BLAST return using the JGI gene model as query.

d. Definition line is from the Arabidopsis accession at TAIR (http://www.arabidopsis.org).

* Transcript level differences confirmed by qRT-PCR.

**Table 2:** Genes up or down-regulated in PCN transgenics involved in hormone related processes.

JGI Gene Accession^a^	Fold Change^b^	Arabidopsis^c^	Definition Line^d^
*fgenesh4_pm.C_LG_II000702	4.67	AT4G25420	gibberellin 20-oxidase
*grail3.0050017401	3.78	AT3G62100	auxin-responsive protein
*estExt_fgenesh4_pg.C_LG_XI0670	3.16	AT1G78440	gibberellin 2-oxidase
*grail3.0061005101	3.08	AT3G62100	auxin-responsive protein
gw1.VI.2253.1	−2.41	AT5G21482	putative cytokinin oxidase
estExt_Genewise1_v1.C_LG_X3745	−3.12	AT3G16770	ethylene-response element binding protein

a. JGI gene accession refers to the accession number assigned by the Joint Genome Institute (http://genome.jgi-psf.org).

b. Fold Change is expressed as the ratio of gene expression in *PCN* gain of function transgenenics to wild type control.

c. Arabidopsis refers to the accession number of the best Arabidopsis BLAST return using the JGI gene model as query.

d. Definition line is from the Arabidopsis accession at TAIR (http://www.arabidopsis.org).

* Transcript level differences confirmed by qRT-PCR.

Nineteen cell wall-related genes have altered transcript levels in 35S::PCN-miRNAd (Table 2). Four of the eight cell wall-related genes with increased transcript levels are involved in cellulose biosynthesis, including the previously mentioned orthologs of *IRX3* and *IRX8* as well as orthologs of CESA1/RSW1 (grail3.0052005901) and CSLA9/RAT4 (estExt_fgenesh4_pm.C_LG_VIII0087). The remaining eleven wall-related genes have lower transcript levels in 35S::PCN-miRNAd and participate in functions including lignification (putative laccase, cinnamoyl CoA reductase, chalcone synthase), and pectin biosynthesis and modification (putative pectin methylesterases and pectinesterase).

Six hormone-related genes are misregulated in 35S::PCN-miRNAd (Table 3). Two putative auxin-responsive genes (grail3.0050017401, grail3.0061005101) show elevated transcript levels in 35S::PCN-miRNAd. While two gibberellin-related genes have higher transcript levels in 35S::PCN-miRNAd, one (GA 20-oxidase, fgenesh4_pm.C_LG_II000702) is putatively involved in GA biosynthesis [42] while the other (GA 2-oxidase, estExt_fgenesh4_pg.C_LG_XI0670) is putatively involved in GA catabolism [43]. Transcript abundance for a gene encoding a putative cytokinin oxidase (gw1.VI.2253.1) involved in inactivation of cytokinin [44] and a gene encoding a cytokinin response factor (Table 1, gw1.I.8677.1) are lower in 35S::PCN-miRNAd. Auxin, cytokinin, ethylene, and gibberellins have been implicated in various studies as regulators of vascular development and secondary growth, although their precise roles remain uncertain [1].

**Table 3:** Genes up or down-regulated in PCN transgenics involved in cell wall synthesis related processes.

JGI Gene Accession^a^	Fold Change^b^	Arabidopsis^c^	Definition Line^d^
*estExt_Genewise1_v1.C_LG_VI2188	3.76	AT5G17420	cellulose synthase, CESA7/IRREGULAR XYLEM 3 (IRX3)
estExt_fgenesh4_pm.C_LG_VIII0087	3.43	AT5G03760	cellulose synthase like, CSLA9/RAT4
grail3.0052005901	3.35	AT4G32410	cellulose synthase, CESA1/RADIALLY SWOLLEN1 (RSW1)
eugene3.00111083	3.10	AT5G54690	galacturonosyltransferase, IRX8
gw1.XV.2531.1	2.89	AT5G61750	germin-like protein-like
fgenesh4_pg.C_LG_I003312	2.82	AT1G23460	polygalacturonase
gw1.VII.534.1	2.72	AT4G28380	extensin-like protein
gw1.VI.781.1	2.44	AT4G30420	nodulin-like protein; MtN21 gene product
gw1.XIII.2619.1	−2.52	AT2G23910	cinnamoyl CoA reductase-like
gw1.131.165.1	−2.55	AT1G56710	polygalacturonase
grail3.0048017501	−2.61	AT1G03870	fasciclin-like arabinogalactan 9 (FLA9)
gw1.856.4.1	−2.83	AT3G22142	proline-rich cell wall protein
estExt_Genewise1_v1.C_LG_VII1401	−2.97	AT5G09760	pectin methylesterase-like protein
gw1.VIII.2100.1	−3.37	AT5G05390	LACCASE 12 (LAC12)
eugene3.00140920	−3.55	AT5G13930	chalcone synthase, TRANSPARENT TESTA 4
estExt_fgenesh4_pm.C_1480004	−3.78	AT2G44480	glycosyl hydrolase family 1
fgenesh4_pg.C_LG_V000014	−3.95	AT5G09760	pectin methylesterase-like protein
gw1.X.3259.1	−4.32	AT3G10720	pectinesterase
gw1.III.2711.1	−6.6	AT1G62500	putative proline-rich cell wall protein

a. Accession number assigned to the assayed gene model by the Joint Genome Institute (http://genome.jgi-psf.org).

b. Fold Change is expressed as the ratio of gene expression in PCN gain of function transgenenics to wild type control.

c. Accession number of the best Arabidopsis BLAST return using the JGI gene model as query.

d. Definition line is from the Arabidopsis accession at TAIR (http://www.arabidopsis.org).

* Transcript level differences confirmed by qRT-PCR.

## Discussion

Class III HD ZIP transcription factors have been implicated in regulating diverse developmental processes, but their function during secondary vascular development has not been addressed. In addition, the genes and cellular processes regulated by Class III HD ZIPs are poorly understood. We examined here the expression and function of a *Populus* Class III HD ZIP, *POPCORONA* (*PCN*), during secondary growth and correlated *PCN* expression with changes in anatomy and gene expression that are consistent with a role in influencing cell differentiation.

Class III HD ZIPs are best characterized in *Arabidopsis thaliana*, where they have been shown to be involved in meristem initiation and function, polarity of lateral organs, and vascular development. Interestingly, among the Class III HD ZIPs in *A. thaliana*, only *REVOLUTA* and *CORONA* (*CNA*) present loss of function phenotypes [10], [11], [45], [46], [47], [48]. The *cna* loss of function phenotype is subtle, and largely limited to a slightly increased meristem size [11]. In *A. thaliana*, *CNA* is among the earliest expressed markers of vascular development [11], [25], and is expressed in procambial cells in leaves, shoot apical meristems, floral meristems, stamens, and carpels [11], [25]. A zinnia ortholog of *CNA*, *ZeHB-13*, is also expressed in procambial cells of developing leaves, but is not expressed at detectable levels in mature leaves [25]. In addition, *ZeHB-13* is expressed in tracheary elements differentiating in vitro, but is not directly induced by cytokinin or auxin in that system [25]. We found that *PCN* is not restricted to provascular cells or primary xylem in *Populus* stems during primary growth. In *Populus*, *PCN* is also expressed during secondary growth, with strongest expression in phloem fibers, the cambial zone, developing xylem, and potentially pith (Figure 3). Interestingly, *PCN* expression is maintained in older secondary xylem, and appears to be associated with rays (Figure 3). The observation that *PCN* expression is maintained in secondary vascular tissues but not primary vasculature of leaves could reflect differences among species, but more likely reflects differences among these tissue types. Specifically, cambial and ray tissues are not present in primary vasculature of leaves.

To examine the function of *PCN*, we created transgenics expressing either an artificial miRNA targeting PCN transcripts (35S::miRNA-PCN) or a *PCN* cDNA with the endogenous miRNA recognition site mutated (35S::PCN-miRNAd). 35S::miRNA-PCN plants showed a subtle phenotype, which includes precocious differentiation of phloem fibers, and abnormal lignification of pith cells in proximity to primary vascular bundles (Figure 6). This subtle phenotype likely reflects the observation that the artificial miRNA was only partially effective in reducing *PCN* transcript levels, but could also reflect functional redundancy with other family members as has been described for *A. thaliana* Class III HD ZIPs [10]. While the *A. thaliana cna* loss of function phenotype is subtle [11], *A. thaliana* plants expressing an antisense *CNA* transgene present a strong phenotype that includes dwarfing, expansion of xylem and interfascicular tissues, and lignification into the pith (Kim et al., 2005). Possible explanations for the discrepancy between these phenotypes include that *cna* alleles characterized may not be complete loss of *CNA* function, or that the sense transgene affected expression of multiple Class III HD ZIP family members.

*Populus* overexpressing a miRNA-resistant (non-cleavable) *PCN* (35S::PCN-miRNAd) had stronger stem phenotypic changes, with delayed differentiation of secondary xylem and severe reduction of phloem fiber differentiation (Figure 6). Cells consistent with nascent phloem fibers form in appropriate positions in 35S::PCN-miRNAd plants, but fail to complete differentiation or fully lignify. Phloem fibers could be considered abaxial in their position within the stem, as their position is analogous to and continuous with the abaxial phloem of primary vascular tissues in leaves. Although one possible interpretation of this phenotype is that overexpression of *PCN* promoted adaxialization of the stem affecting phloem fiber development, we do not favor this interpretation, in part because phloem fibers initiate in an appropriate position, and simply fail to properly complete differentiation. Additional evidence supporting primary affects of *PCN* on cell differentiation vs. patterning was provided by gene expression profiling of *PCN* mutant stems. Genes associated with the biosynthesis, response, or catabolism of auxin, gibberellin, and cytokinin were misregulated in 35S::PCN-miRNAd stems (Table 3). In addition, an AP2-like transcription factor orthologous to *Cytokinin Response Factor 2* (*CRF2*) is downregulated in 35S::PCN-miRNAd stems. *CRF2* is upregulated by cytokinin, and acts with other *CRF*s to regulate a large fraction of genes involved in cytokinin response [49]. Interestingly, these same hormones have all been implicated in fiber differentiation, with auxin and gibberellin flowing basipitally through the stem from leaves [50], [51], and cytokinin from roots [52]. Indeed, mutations in the related *A. thaliana* Class III HD ZIP *REVOLUTA*/*INTERFASCICULAR FIBERLESS 1* can result in defects in interfascicular fiber differentiation [47], which are associated with polar auxin transport defects in stems [48]. These results are also consistent with the implication of Class III HD ZIP genes in affecting expression of *PIN1*, an auxin efflux transporter, during embryogenesis in *A. thaliana* [30]. Thus, we favor the interpretation that *PCN* phenotypes do not reflect patterning or polarity defects, but more likely hormone-related defects and changes in genes involved in cell differentiation and cell wall biosynthesis, as discussed below.

In addition to hormone-related genes discussed above, other transcription factors as well as cell wall biosynthetic genes are misexpressed in 35S::PCN-miRNAd stems (Table 1, 2). Interestingly, these genes include homologs of *SECONDARY CELL WALL-ASSOCIATED NAC DOMAIN* (*SND*), *VASCULAR RELATED NAC DOMAIN* (*VND*), and *MYB*s that have been implicated in a hierarchical pathway regulating genes involved with tracheary element development and cell wall synthesis and lignification [9], [39], [40], [41]. Cell differentiation and cell wall-related genes associated with these transcriptional regulators also show misregulation in 35S::PCN-miRNAd (see Results). The changes in cell wall–related genes, including downregulation of lignin-related genes in 35S::PCN-miRNAd stems are consistent with anatomical phenotypes, which include inhibition of phloem fiber lignification in 35S::PCN-miRNAd and abnormal lignification of cells in the pith of 35S::miRNA-PCN. Importantly, it should be noted that the two SND/VND genes misexpressed in 35S::PCN-miRNAd are orthologous to SND2 and NAC7/VND4, not the better characterized SND1 and VND6&7 [8], and the expression relationships of MYBs to putative targets is altered. Thus, in addition to differences expected between species and tissue types, the relationships among the transcriptional networks underlying secondary growth in *Populus* and primary growth in *A. thaliana* will require further study to comprehensively compare.

Given that Class III HD ZIPs are evolutionarily ancient and predate evolution of vascular tissues [29], the function of *PCN* during secondary growth must be derived. *PCN* and its orthologs in *A. thaliana* and *Zinnia* [11], [17], [25] are not uniquely expressed during secondary vascular development, and the proteins’ biochemical functions are presumably the same in the different tissues where it is expressed. However, it is possible that target genes of *PCN* vary between tissues, because of differences in available partners for dimerization, dominant negative LITTLE-ZIPPERs [53], [54], different co-factors, or differences in regulation by miRNA165/166 in different tissues. Although beyond the scope of the work presented here, it will ultimately be very informative to examine changes in gene expression for *PCN* or related mutants in the various tissues in which these genes are normally expressed. Unfortunately there are no published reports of gene expression profiling for *A. thaliana CNA* mutants to date. An example of a potentially important difference for *PCN*/*CNA* function among tissues is illustrated by the observation that *cna* mutations dramatically enhance *CLAVATA* (*CLV*) mutant phenotypes in *A. thaliana*, and result in misexpression of *CLV3* and *WUSCHEL* [11]. Interestingly, microarray profiling of gene expression in *Populus* stems undergoing secondary growth revealed that *Populus* orthologs of *CLV3* and *WUS* are not expressed during secondary growth [5]. Although *PCN* may interact with other family members related to *WUS* and *CLV3* in the cambium, this nonetheless indicates significant differences for *PCN* function during primary and secondary growth.

## Materials and Methods

### Phylogenetic Analysis of Class III HD Zips

Gene sequences from the Class III HD Zips gene family were recovered from species that have complete genome sequence using the BLAST function in phytozome.net (Table S1). Genome databases searched included *Arabidopsis lyrata, Brachypodium distachyon, Cucumis sativus, Glycine max, Manihot esculenta, Mimulus guttatus, Ricinus communis, Selanginella moellendorffii, Zea mays Arabidopsis thaliana*, *Carica papaya*, *Clamydomonas reinhardtii, Medicago truncatula*, *Oryzas sativa*, *Populus trichocarpa*, *Physcomitrella patens, Sorghum bicolor*, and *Vitis vinifera*. 94 unique sequences were found from the 18 complete species genomes. The sequences were aligned with known Class III HD Zips from previous work for identification (Prigge & Clark, 2006; Floyd *et al.,* 2006). For *Z. mays* three additional sequences were found and in *M. truncatula* one additional sequence was identified. *POPCORONA* (*PCN*) is variously known as Pt-ATHB.12; Joint Genome Institute Populus v.1.1 gene model fgenesh4_pm.C_LG_I000560: Phytozome Populus v2.0 gene model POPTR_0001s18930.

Nucleotide sequences were translated into amino acid sequences using DAMBE [55]. Amino acid alignment was achieved using ClustalX (version 1.8) [56] with all default settings. The alignment was modified manually using MacClade 3.08 [57] and exported in NEXUS format for further analysis with the final alignment containing 3261 sites. A nucleotide alignment was created based on the amino acid alignment using DAMBE [55].

Maximum parsimony analysis of the nucleotide alignment was performed using PAUP* version 4.0b10 for Macintosh [58], with heuristic searches using the TBR branch-swapping algorithm and 1000 random taxon addition replicates; the maxtrees setting was allowed to increase automatically as necessary. Gaps within the alignment were treated as missing data. Relative support for clades was assessed using 1000 bootstrap replicates with 10 random taxon addition replicates per bootstrap replicate and maxtrees capped at 100. Bayesian analyses, using the GTRIG model of sequence evolution as selected by MrAIC [59], were implemented in MrBayes 3.1.1 [60]. Double analyses were run with four chains for 1,000,000 generations, sampling every 100 generations. Trees from the first 250,000 generations (2,500 trees) from each run were discarded as burn-in, following the authors’ recommendations and consistent with the observation that the likelihood scores from both runs had stabilized. The sampled trees from both analyses were pooled and majority-rule consensus trees were constructed from the resulting 15,000 trees in order to estimate Bayesian clade credibility values.

### Plant Cultivation and Transformation

Hybrid aspen clone INRA 717-IB4 (*Populus alba × P. tremula*) was used for all experiments. Plants were propagated and transformed using previously published methods [61]. Two independently transformed lines were used for *PCN* gain of function analysis (35S::PCN-miRNAd 2-1 and 35S::PCN-miRNAd 2-3). Two independently transformed lines were used for *PCN* knock down analysis (35S::miRNA-PCN 6-1 and 35S::miRNA-PCN 6-4). All experiments were repeated at least twice using each of the above transformed lines and matched wild-type controls with similar results, unless otherwise stated.

### Whole Mount *in situ* Hybridization

Whole mount *in situ* hybridization was performed as previously described [7]. A 220-bp fragment from the 5′ end of the *PCN* coding region and a 292-bp fragment from the 5′ end of the *Pop50S* coding region were selected to design primers to generate the template of probes using the gene-specific primers:. PtCN-F:5′CTTCTGGTTGTTGCGTTATAC-3′; PtCN-R:5′ CTCGGGCCATTTTGAGTATTT-3′. Pop50S-F:5′CCTAGTGTTCCTGTAACTCGCATTGG-3′; Pop50S-R:5′CTCCCACCACCATGTTGTCCGTAAGTG-3′. T7 promoter sequence 5′-TAATACGACTCACTATAGGG was added to the 5′ end of the PCN-R primer and Pop50S-R primer sequence to generate templates of antisense probes for *PCN* and *pop50S*. T7 promoter sequence was added to the 5′ end of PCN-F primer sequence and Pop50S-F primer sequence to generate templates of sense probes for *PCN* and *pop50S*.

### Recombinant DNA Constructs

The overexpression of Class III HD Zip genes is not expected to yield strong phenotypes, as these genes contain a microRNA binding site which negatively regulates transcript levels. To prevent this mechanism masking the effects of overexpression of the gene of interest, the microRNA binding site of *PCN* was changed to abolish miRNA recognition while leaving the protein sequence unchanged, as previously described [13]. The coding sequence of *PCN* was amplified with PCR primers which replaced base pairs in the microRNA binding site *PCN*_F 5′-CTGGAATGAAGCCTGG**a**CC**a**GATTCCAG-3′ and *PCN*_R 5′-GCAACGATTCCACTGGAATC**t**GG**t**CCA-3′ and sub-cloned into vector ART7 to make the entry clone ART7-*PCN*. The insert was recombined into ART27 to generate 35S::PCN-miRNAd.

The 35S::miRNA-PCN construct was assembled to drive expression of a synthetic miRNA by the 35S promoter. A 21 nucleotide sequence (5′ TTGCGTTATACTTCTGTTTTA 3′) specific to *PCN* and its paralog, Pt-ATHB.11 (Phytozome *Populus* v2.0 gene model POPTR_0003s04860; Joint Genome Institute *Populus* v1.1 gene model estExt_fgenesh4_pg.C_LG_III0436) was targeted based on published targeting parameters [62], [63], [64] and uniqueness to *PCN* and its paralog, Pt-ATHB.11. The exact complementary sequence (miRNA) is 5′ TAAAACAGAAGTATAACGCAA. Mismatches were introduced at positions four, nine, and ten, and in positions 20 and 21 in the complementary strand, to produce the following miRNA and miRNA* sequences.

miRNA: 5′ TAAAACAGAAGTATAACGCAA 3′

miRNA*: 5′ ACGCGTTATACAACTGTATTA 3′

A DNA was synthesized with these sequences replacing the normal miRNA and miRNA* sequences within MIR164b [62] to produce the following sequence containing Xho and Xba restriction sites at the 5’ and 3’ ends, respectively. The sequence CACC was added at 5’ end for directional cloning:

Xho-Xba MIR-PCN

5’CACCCTCGAGGAGAATGATGAAGGTGTGTGATGAGCAAGATAAAACAGA

AGTATAACGCAATTACTAGCTCATATATACACTCTCACCACAAATGCGTGTAT

ATATGCGGAATTTTGTGATATAGATGTGTGTGTGTGTTGAGTGTGATGATATG

GATGAGTTAGTTCACGCGTTATACAACTGTATTATCATGACCACTCCACCTTG

GTGACGATGACGACGAGGGTTCAAGTGTTACGCACGTGGGAATATACTTATA

TCGATAAACACACACGTGCGTCTAGA3’

### Microarray Analysis and Q-RT-PCR

For each genotype, three bulks of three plants each were combined for RNA isolation from shoot apices, leaves, stem, and root tissue of two-month-old tissue culture trees for microarray analysis and Q-RT-PCR. Affymetrix GeneChip® Poplar Genome Array oligonucleotide microarrays were used for all microarray hybridizations. For microarray hybridizations, total RNA was isolated from entire, defoliated stems of two months old tissue culture grown plants. Three independent biological replicate RNAs were isolated for each of three overexpression and miRNA lines, and four independent biological replicates of matched wild-type controls. Total RNA quantity and quality was determined using an Agilent Bioanalyzer (Agilent Technologies, USA). Biotin labeling of target RNAs was performed with one-cycle target kit (One-Cycle Target Labeling and Control Reagents, Affymetrix, P/N 900493), and hybridized according to the manufacturer’s protocol.

Analysis of microarray data was performed with dChip (http://biosun1.harvard.edu/complab/dchip/). Data were normalized across arrays using median probe intensity of the baseline array. Preliminary analysis established filtering and statistical cutoff thresholds, using as criteria the false discovery rate, the identification of biologically meaningful genes, and the inclusion of genes confirmed as being misexpressed by Q-RT-PCR as primary criteria. In the final analysis, model-based expression data were filtered by removing genes whose representative probes did not exceed > =  40% (presence call %) on a given array and > =  50% among arrays. Filtered genes were then compared based on fold expression difference and t-test (p-value of 0.05). False discovery rate was determined by 50 permutations to be 2.4% in the final analysis. MIAME-compliant information about samples, array platform, microarray data and further details of the samples are available through NCBI GEO (**GSE19467**). Output from microarray analysis is shown at the probe-level in Table S2 (and includes probes designed against *Populus* ESTs), and for *P. trichocarpa* gene models in Table S3.

Gene expression differences estimated by microarray analysis were confirmed using qRT-PCR for the indicated genes in Tables 1, 2 and 3. Gene-specific PCR primers were designed to target genes showing differential expression in the microarray comparison of 35S::PCN-miRNAd, 35S::miRNA-PCN and wild-type trees. Primers with Tm of >59°C were designed to produce a product 200-300bp. A tubulin-encoding gene (JGI accession estExt_fgenesh4_pm.C_LG_III0736) was used as reference gene for Q-RT-PCR. Q-RT-PCR was performed with an MJ Mini Opticon (BioRad) following the manufacturer’s protocols.

## Supporting Information

Table S1 Gene sequences from the Class III HD Zips gene family recovered from species that have complete genome sequence using the BLAST function in phytozome.net.
(TXT)

Table S2 Output from microarray analysis at the probe-level, including probes designed against *Populus* spp. ESTs.
(XLS)

Table S3 Output from microarray analysis at the level of *P. trichocarpa* gene models, excluding probes designed against *Populus spp.* ESTs.
(XLS)
